# Effects of whole-body vibration training combined with KAATSU training on lower limb joint muscle strength in older women

**DOI:** 10.3389/fphys.2023.1231088

**Published:** 2023-08-29

**Authors:** Weizhi Xiong, Xuefeng Liu

**Affiliations:** ^1^ School of Physical Education, Chengdu Sport University, Chengdu, China; ^2^ Physical Rehabilitation Center, Sichuan Sports College, Chengdu, China

**Keywords:** whole-body vibration training, Kaatsu training, elderly, Equivalent speed, muscle strength

## Abstract

**Objective:** This study aimed to investigate the effect of whole-body vibration training (WBVT) combined with KAATSU training (KT) on lower limb joint muscle strength and to provide a reference for improving muscle strength in older women.

**Methods:** A total of 86 healthy older people was randomly divided into WBVT group (WG, *n* = 21), KT group (KG, *n* = 22), combined intervention group (CIG, *n* = 20) and control group (CG, *n* = 23). WG and CIG subjects underwent WBVT, and KG and CIG subjects underwent 150 mmHg and lower limb joint and local compression intervention for 16 weeks (three times per week, about 15 min/time). The peak torque (PT) and endurance ratio (ER) of joint flexion or extension were tested for all subjects.

**Results:** 1) Results at 16 weeks were compared with the baseline data. The knee extension and ankle flexion PT (60°/s) in CIG increased by 14.3% and 15.3%, respectively (*p* < 0.05). The knee extension PT (180°/s) increased by 16.9, 18.4% and 33.3% in WG, KG and CIG (*p* < 0.05), respectively, and the ankle extension PT (180°/s) in CIG increased by 31.1% (*p* < 0.05). The hip, knee extension and ankle flexion ER increased by 10.0, 10.9% and 5.7% in CIG (*p* < 0.05), respectively. 2) Results were compared among groups at 16 weeks. The relative changes were significantly lower in WG, KG and CG compared to CIG in the knee extension and ankle flexion PT (60°/s) (*p* < 0.05). The relative changes were significantly greater in WG, KG and CIG compared to CG in the knee extension PT (180°/s) (*p* < 0.05). The relative changes were significantly lower in WG, KG and CG compared to CIG in the ankle extension PT (180°/s) (*p* < 0.05). The relative changes were significantly lower in WG, KG and CG compared to CIG in the hip extension ER (*p* < 0.05). The relative changes were significantly lower in CG compared to CIG in the knee extension ER (*p* < 0.05).

**Conclusion:** Sixteen-week WBVT and KT increased the knee extensor strength in older women. Compared with a single intervention, the combined intervention had better improvements in the knee extensor and ankle flexor and extensor strength and hip extension muscle endurance. Appears to be some additional benefit from combined intervention above those derived from single-interventions.

## 1 Introduction

Ageing-induced decline in human muscle strength increases the risk of falling in the elderly, so delaying such decline is an important strategy to prevent falls (Sugaono et al., 2020; Kim et al., 2022). Whole-body vibration training (WBVT) (Van et al., 2021) is suitable for people with poor motor function or those who are unaccustomed to active exercise; it includes muscle strengthening exercises (Alam et al., 2018; Cheng et al., 2021a). Compared with interventions without vibration, WBVT can stimulate the muscle group in a vibration site and engage more muscle fibres in muscle contraction, thereby effectively improving the strength of muscles in the joint (Pescatello et al., 2009; Fragala et al., 2019; Sugaono et al., 2020). WBVT (3 weeks, frequency 35–40 Hz, amplitude 4 mm) can enhance the maximal voluntary isometric contraction of knee extensors in older adults with knee osteoarthritis (Simão et al., 2019). Six weeks of WBVT (frequency 30 Hz, amplitude 3.9 mm) improved lower extremity joint muscle strength in older adults (Perchthaler et al., 2015). Another study found that high-frequency WBVT (24 weeks, 40–45 Hz, 3 mm amplitude) was more effective in improving knee and ankle joint strength in older women than low-frequency WBVT (10–15 Hz) (Li and Cheng, 2018). However, changes in muscle endurance have not been analysed yet. KAATSU training (KT), also known as blood flow restriction training, is causes muscle ischaemia in the distal limb through compression, stimulates muscle growth and improves muscle fitness at a relatively low exercise intensity (Golubev et al., 2020; Ishizaka et al., 2022). KT can effectively improve muscle strength and reduce the risk of injury (Karabulut et al., 2010; Golubev et al., 2021). Pressure and strength should be reasonably set in KT to prevent safety problems (Nakajima et al., 2011). About 10% of 1 RM intensity is the minimum intensity that stimulates muscles to produce hypertrophy, which is closely related to the degree of muscle activation and muscle cell swelling. Low-intensity resistance training (20%–30% 1 RM) combined with KT, which focuses on limb and trunk muscles, induces muscle hypertrophy and enhances muscle strength (Abe et al., 2010).

The two training methods (WBVT and KT) can promote local muscle blood flow and muscle metabolic stress level and allow the use of low weight bearing to increase muscle strength (Kim et al., 2016; Cheng et al., 2022). Whether WBVT and KT (Combination Intervention) can improve muscle strength in the elderly remains unclear. Therefore, the current study targeted healthy older women and conducted a 16-week WBVT and KT intervention to explore the effect of their combination on muscle strength in lower limb joints in older women. Results will provide a basis for improving muscle strength in the elderly and prevent muscle strength loss caused by ageing. We hypothesise that combined intervention has a better effect on lower limb joint muscle strength in older women than a single intervention.

## 2 Materials and methods

### 2.1 Participants

This study was approved by the Sichuan Sports College Ethics Committee for Human Testing (No: 202210). Through visitation and advertisements, we recruited healthy older women who were willing to exercise in large parks and communities around our school. The inclusion criteria were as follows: age of 60–69 years; willingness to exercise; passed physical examination for cardiovascular diseases; and compliance with the Declaration of Helsinki and signed informed consent. The exclusion criteria were as follows: diagnosis of endocrine metabolic and chronic diseases (such as diabetes, heart disease and stroke), osteoporosis, motor dysfunction, epilepsy and motor neuron diseases; consumption of testosterone supplements and drugs affecting bone metabolism; and engagement in other regular exercises.

**FIGURE 1:**
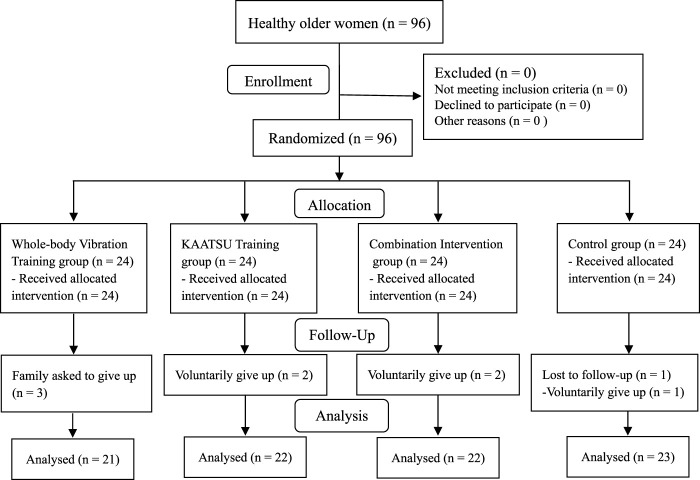
Participant selection flow diagra.

This study considered a four-group and pretest-protest experimental design, with a sample dropout rate of approximately 12% based on the previous results of WBVT (Perchthaler et al., 2015; Cheng et al., 2021b) or KAATSU training (Yasuda et al., 2014a; Letieri et al., 2018) performed in older women. The G-power software was used to calculate with an effect size of 0.3, a power of 0.8 and an α value of 0.05, and a sample size of at least 96 would be needed. In the initial stage of the project, 96 subjects met the requirements and were randomly divided into WBVT group (WG), KAATSU training group (KG), combination intervention group (CIG, WBVT and KAATSU training) and control group (CG). Ten subjects withdrew because of personal or family reasons in the early, middle and late experimental stages, with a sample attrition rate of 10.4%. Finally, 86 subjects completed the entire experimental procedure (Figure 1; Table 1). One-way ANOVA showed no significant difference in baseline data among the groups (*p* > 0.05).

**TABLE 1:** Basic information of the study participants.

Group	n	Age(years)	Height(cm)	Body mass(kg)	BMI(kg/m2)
WG	21	64.7 ± 1.8	157.3 ± 1.9	64.2 ± 5.7	25.9 ± 2.3
KG	22	65.1 ± 1.9	158.1 ± 2.5	63.7 ± 4.8	25.5 ± 1.9
CIG	20	64.3 ± 2.2	157.7 ± 3.1	63.8 ± 4.9	25.7 ± 2.5
CG	23	64.6 ± 2.0	158.3 ± 2.4	64.1 ± 5.0	25.6 ± 2.2
*F*		0.585	0.749	0.040	0.163
*P*		0.627	0.526	0.989	0.921

Note: WG: Whole-body vibration training group, KG: KAATSU, training group, CIG: combination intervention group, CG: control group, BMI: body mass index.

### 2.2 WBVT

A previously described protocol of WBVT was used (Cheng et al., 2022). A mechanical vibration frequency of 45 Hz and an amplitude of 3 mm are beneficial to improve the strength of human joint muscles. WBVT was performed on WG and CIG subjects for 16 weeks (frequency: three times per week, single time: about 15 min per time). A US Power-Plate vibration trainer (American model Pro5 AIR™, vibration 106 frequency range 25–50 Hz, amplitude range 1–3 mm, 107 maximum weight 250 kg) was used. Participants in WG and CIG completed three actions (each movement was completed in three groups, and each group was completed 10 times) when the vibrator was on. Half squats (the knee joint angle is about 90°, 20 s/set with an interval of 10 s), static weight-free squats (20 s/set with an interval of 10 s) and left and right lunge squats (10 times/set with a 20-completed with the vibrator on second rest between sets) were conducted when the vibrator was on (Cheng et al., 2022). Participants in CG completed the same actions for the same number of repetitions and durations as those in WG and CIG, but the device was turned off (Cheng et al., 2022). The whole experiment was supervised by professionals to ensure the safety and quality of the training.

### 2.3 KAATSU training

Previous studies showed that 5–14 cm-wide pressure band and 110–200 mmHg pressure can exert good training effects and few negative effects (Kim et al., 2016; Luebbers et al., 2019). In this regard, KG and CIG subjects used a pressure trainer (KAATSU CYCLE MASTER, JAPAN) to pressurise the left and right legs, quadriceps, biceps and gastrocnemius at 150 mmHg (compression band length 114.5 cm, 5.5 cm wide, 0.4 cm thick. cycle: 16 weeks, frequency: three times per week, single time: about 15 min per time). The same four-movement training programs as WG, CIG and CG were performed, and the experimenter guided the movement and controlled the intensity.

### 2.4 Isokinetic muscle strength test

Isokinetic muscle strength test with bilateral hip, knee and ankle (The subjects perform 5 reptitions at 60° per second and 25 reptitions at 180 degress per second) before and after 16 weeks. Among them, the hip and ankle subjects were in the decubitus position and the knee position. The ranges of motion of the hip, knee and ankle joint were 120, 80° and 70°, respectively (Cheng and Jiang, 2020).

Isokinetic muscle strength was tested using peak torque (PT) and muscle endurance. PT refers to the maximum output moment (Nm) produced by muscle contraction during whole joint activity and reflects the strength of a subject (Cheng and Jiang, 2020). Endurance ratio (ER) is ability to tolerate fatigue during repeated contraction of the joint muscles. ER is calculated as follows: (the sum of the total work done at the 21st time—the 25th time) to (the sum of the total work done at the first—the fifth time) in the 180°/s test. It reflects a subject’s muscle endurance level, and values close to 1 indicate enhanced endurance (Cheng and Jiang, 2020).

### 2.5 Mathematical statistics

The mean values and standard deviations of the PT and ER of the hip, knee and ankle (averaged left and right data of each subject) were calculated using SPSS Statistics 20.0 statistical software. The normality of the data was measured using Shapiro–Wilk test, and the data were transformed if they did not fit within the normal distribution. The experimental design for the groups (four groups) considered time (two measurements) (Cheng et al., 2021a). Two-factor ANOVA of time and group interaction was also conducted. When an interaction was observed, whether a separate effect of time or group occurred was determined. When no interaction was observed, whether a substantial effect occurred was determined. Post-hoc multiple comparisons were performed using Bonferroni correction, and the significance level (α) was set as 0.05.

## 3 Results

**FIGURE 2:**
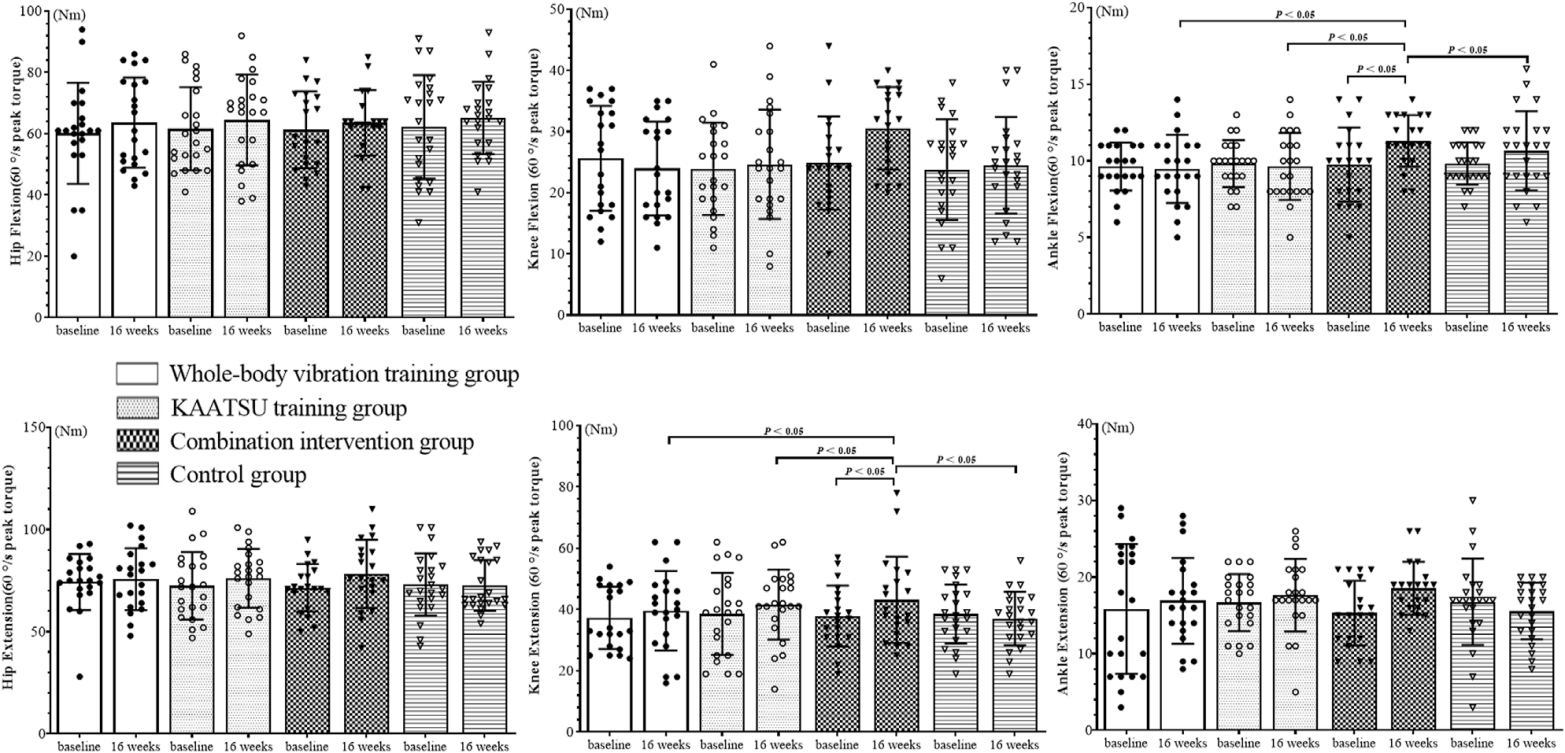
Results of 60°/s peak torque test of subject lower limb joints.

**FIGURE 3:**
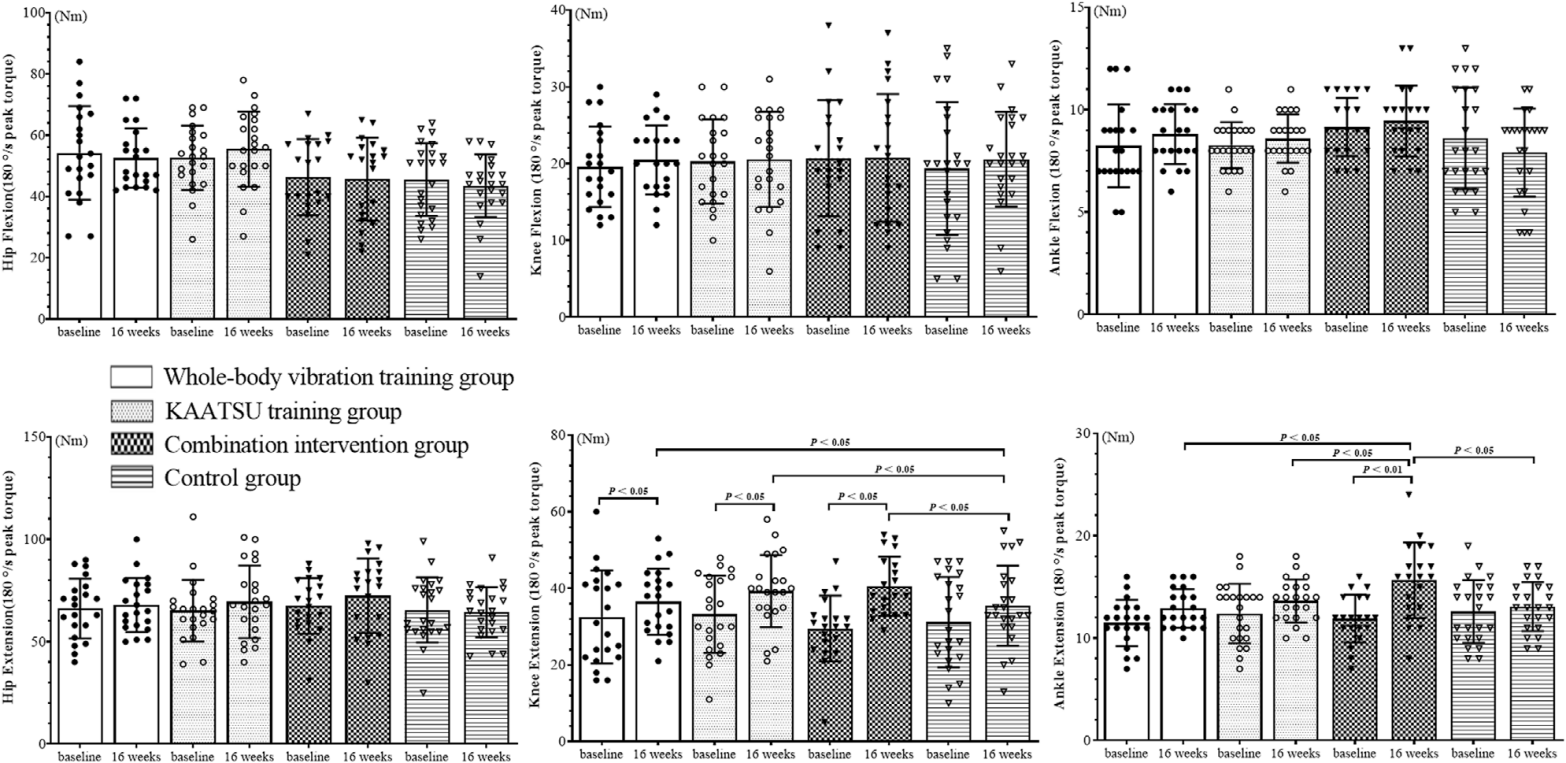
Results of 180°/s peak torque test of subject lower limb joints.

**FIGURE 4:**
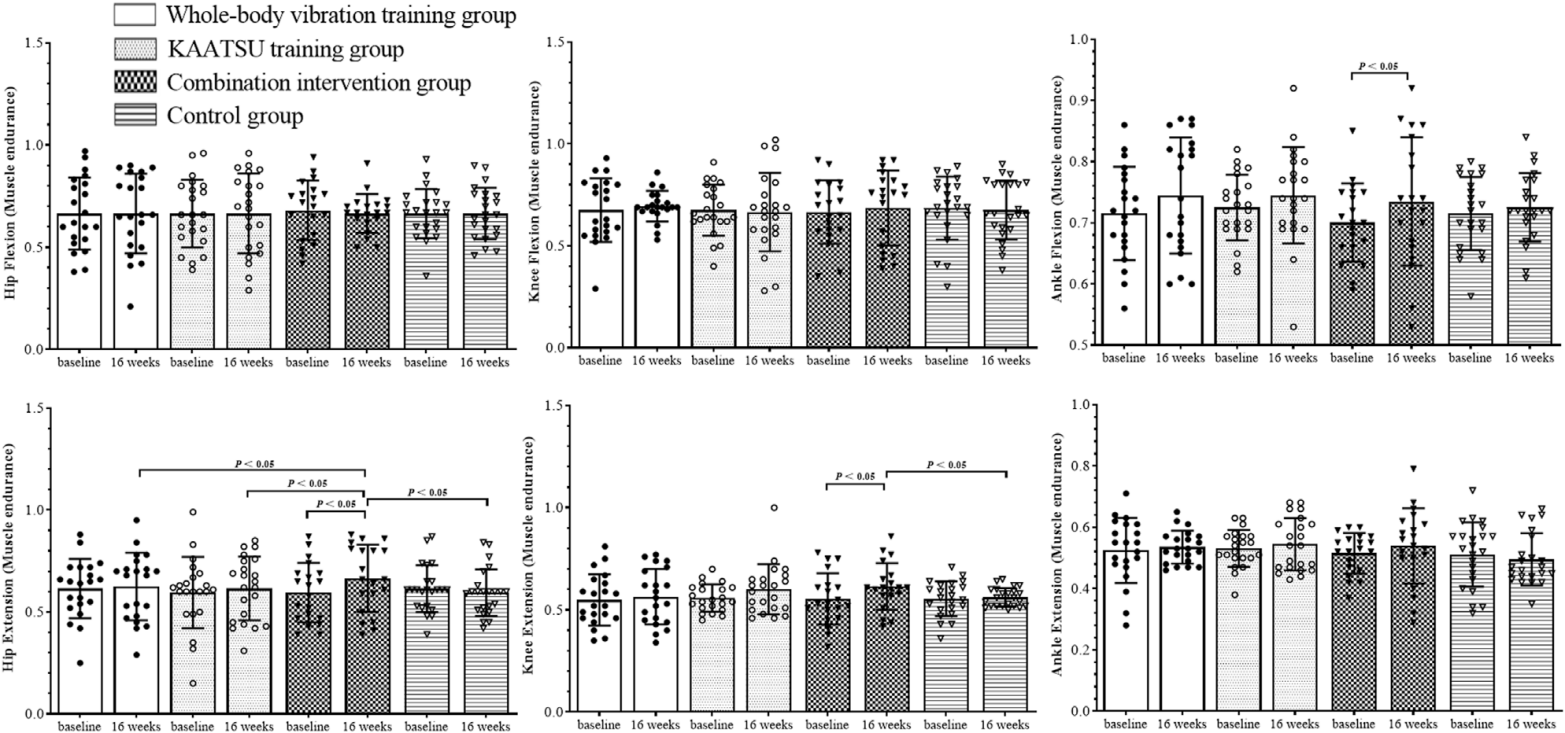
Results of muscle endurance test of subject lower limb joints.

The isokinetic muscle strength of the lower limbs was tested (Figures 2–4). The Shapiro–Wilk test indicated that the data had a normal distribution (*p* > 0.05). No significant difference in baseline data was found among the groups (*p* > 0.05).

### 3.1 PT (60°/s)

The 60°/s results for hip flexion (F_(3, 164)_ = 0.013, *p* = 0.998) and extension (F_(3, 164)_ = 0.494, *p* = 0.687), knee flexion (F_(3, 164)_ = 1.551, *p* = 0.203) and extension (F_(3, 164)_ = 0.655, *p* = 0.581), ankle flexion (F_(3, 164)_ = 1.351, *p* = 0.260) and extension (F_(3, 164)_ = 1.333, *p* = 0.266) were obtained. No interaction was observed between time and group. The main effects of time or group were further analysed.

Main effects for time: The main effects of time were insignificant on hip flexion (F = 1.776, *p* = 0.184), hip extension (F = 1.650, *p* = 0.201), knee flexion (F = 1.264, *p* = 0.263) and ankle extension (F = 1.630, *p* = 0.203). However, the main effects on knee extension (F = 5.729, *p* = 0.020, η^2^ = 0.169) and ankle flexion (F = 6.267, *p* = 0.013, η^2^ = 0.237) were significant. Comparison with baseline data showed that the knee extension PT increased by 14.3% and ankle flexion PT increased 15.3% in CIG at 16 weeks (*p* < 0.05).

Main effects for groups: The main effects of groups were insignificant for hip flexion (F = 0.133, *p* = 0.941) and extension (F = 0.204, *p* = 0.893), knee flexion (F = 1.834, *p* = 0.143) and ankle extension (F = 0.347, *p* = 0.791). However, the main effect was significant on knee extension (F = 8.557, *p* < 0.001, η^2^ = 0.319) and ankle flexion (F = 4.411, *p* = 0.005, η^2^ = 0.375). Post-hoc differences were found among the groups at 16 weeks. The relative changes were significantly lower in WG, KG and CG compared to CIG in the knee extension and ankle flexion PT (60°/s) (*p* < 0.05).

### 3.2 PT (180°/s)

The 180°/s results for hip flexion (F_(3, 164)_ = 0.372, *p* = 0.773), hip extension (F_(3, 164)_ = 0.373, *p* = 0.772), knee flexion (F_(3, 164)_ = 0.072, *p* = 0.975), knee extension (F_(3, 164)_ = 1.079, *p* = 0.359), ankle flexion (F_(3, 164)_ = 1.122, *p* = 0.342) and ankle extension (F_(3, 164)_ = 2.066, *p* = 0.093) were obtained. No interaction was found between time and group.

Main effects for time: The main effects of time on hip flexion (F = 0.037, *p* = 0.848), hip extension (F = 1.130, *p* = 0.289), knee flexion (F = 0.361, *p* = 0.549) and ankle flexion (F = 0.209, *p* = 0.648) were not significant. However, the effects on knee and ankle extension (F = 17.096, *p* < 0.001, η^2^ = 0.394; F = 18.255, *p* < 0.001, η^2^ = 0.400) were significant. The knee extension PT increased by 16.9, 18.4% and 33.3% in WG, KG and CIG, respectively (*p* < 0.05), and the ankle extension PT in CIG increased by 31.1% (*p* < 0.01) compared with the baseline data.

Main effects for groups: The main effects of groups were insignificant on hip flexion (F = 0.122, *p* = 0.947), hip extension (F = 0.781, *p* = 0.506), knee flexion (F = 0.122, *p* = 0.947) and ankle flexion (F = 2.499, *p* = 0.061). However, the main effects on knee and ankle extension (F = 7.269, *p* < 0.001, η^2^ = 0.117; F = 2.806, *p* = 0.041, η^2^ = 0.149) were significant. Post-hoc differences were found among the groups at 16 weeks. The relative changes were significantly greater in WG, KG and CIG compared to CG in the knee extension PT (180°/s) (*p* < 0.05). The relative changes were significantly lower in WG, KG and CG compared to CIG in the ankle extension PT (180°/s) (*p* < 0.05).

### 3.3 ER test

ER test was conducted on hip flexion (F_(3, 164)_ = 0.025, *p* = 0.995), hip extension (F_(3, 164)_ = 0.667, *p* = 0.574), knee flexion (F_(3, 164)_ = 0.135, *p* = 0.939), knee extension (F_(3, 164)_ = 0.6555, *p* = 0.646), ankle flexion (F_(3, 164)_ = 0.226, *p* = 0.878) and ankle extension (F_(3, 164)_ = 0.409, *p* = 0.746). No interaction was found between time and group.

Main effects for time: The main effects of time on hip and knee flexion (F = 0.028, *p* = 0.867; F = 0.046, *p* = 0.549) and ankle extension (F = 0.341, *p* = 0.560) were not significant. However, the effects on hip and knee extension (F = 5.789, *p* = 0.036, η^2^ = 0.179; F = 4.089, *p* = 0.043, η^2^ = 0.207) and ankle flexion (F = 4.223, *p* = 0.041, η^2^ = 0.325) were significant. The hip and knee extension ER increased by 10.0% and 10.9% and the ankle flexion ER increased by 5.7% in CIG compared with the baseline data, and the differences were statistically significant (*p* < 0.05).

Main effects for groups: The main effects of groups were insignificant on hip and knee flexion (F = 0.028, *p* = 0.994; F = 0.075, *p* = 0.973) and ankle flexion (F = 0.504, *p* = 0.680) and extension (F = 1.273, *p* = 0.286). However, the effects on hip and knee extension (F = 6.287, *p* = 0.006, η^2^ = 0.089; F = 9.685, *p* < 0.001, η^2^ = 0.116) were significant. Post-hoc differences were found among the groups at 16 weeks. The relative changes were significantly lower in WG, KG and CG compared to CIG in the hip extension ER (*p* < 0.05). The relative changes were significantly lower in CG compared to CIG in the knee extension ER (*p* < 0.05).

## 4 Discussion

This study conducted 16-week WBVT and KT interventions in older women to explore changes in the strength and muscle endurance of the flexor and extensor muscle groups of the lower limb joints and determine whether WBVT and KT can improve muscle strength. This study tested the hypothesis that the combined intervention has a better effect on lower limb joint muscle strength in older women than a single intervention.

### 4.1 PT

The knee extension (60°/s and 180°/s) increased by 14.3% and 33.3%, respectively, and the ankle flexion (60°/s) and extension (180°/s) increased by 15.3% and 31.1%, respectively, in CIG. The knee extension PT (180°/s) increased by 16.9% and 18.4% in WG and KG, respectively. Comparison among groups at 16 weeks showed that CIG decreased the knee extension compared with WG, KG and ankle flexion (60/s), suggesting that a single intervention (WBVT or KT) increased knee extensor strength in older female subjects but had no significant effect on ankle strength. Appears to be some additional benefit from combined intervention above those derived from single-interventions. The combined intervention is more effective in improving the muscle strength of knee extensor and ankle flexor and extensor in older female subjects than a single intervention.

On the one hand, WBVT increases the length of the muscle fibres in the muscle spindle and increases the frequency and intensity of nerve pulses, enabling more motor units to participate in a movement (Rittweger, 2010; Marin et al., 2014; Costantino et al., 2018; Oliveira et al., 2021). When WBVT is performed in the human standing posture, the motor unit of the lower limb muscle group is activated and promotes the contraction function of the tendon (Gomes et al., 2019; de Paula et al., 2021; Gonçalves et al., 2023). Compared with no-vibration training, more muscle fibres are stimulated to participate in the contraction, and repeated stimulation improves muscle strength (Pescatello et al., 2009; Fragala et al., 2019; Sugaono and Tecchio, 2020). In addition, WBVT increases the nerve reflex, muscle temperature, blood flow and vibrational resistance in a vibration site, thereby improving neuromuscular performance (Alvarez et al., 2017). WBVT also improves muscle strength. In the present study, we found that WBVT improved PT for knee extension, consistent with previous results (Perchthaler et al., 2015; Simão et al., 2019).

On the other hand, KT restricts blood flow in a pressure site, hindering muscle lactate from diffusing to the blood. Hence, the lactate levels in the muscles considerably increase and cause an acidic environment, which facilitates the stimulation of the pain nerve and protein synthesis and promotes the secretion of insulin-like growth factor-1 (IGF-1) in tissues. The expression of IGF-1 is the main physiological mechanism that promotes muscle strength and hypertrophy (Abe et al., 2005). Letieri et al. (2018) reported that 6 weeks of KT can increase the PT of the knee extensors in the elderly. Yasuda et al. (Yasuda, Fukumura, Fukuda, et al., 2014a; Yasuda, Fukumura, Sato, et al., 2014b) conducted two studies and found that 12 weeks of KT can improve the knee extensor strength in older adults. Some maintenance effects were observed 12 weeks after the cessation of KT (compared with the baseline data) The present study found that KT increased the PT of knee extensors in older subjects, consistent with previous studies (Yasuda et al., 2014b; Letieri et al., 2018). In addition, falls among elderly are largely associated with lower limb muscle strength, and ankle extensor muscle is the best predictor of falls, followed by knee joint (Pijnappels et al., 2008). This study found that the combined intervention improved ankle strength, leading to a positive effect on fall prevention.

The elderly are more likely to suffer from falls in emergency situations because they have reduced explosive force (Sugaono and Tecchio, 2020). However, the recruitment of fast muscle fibres during KT is facilitated by a hypoxic environment caused by metabolite accumulation, which is unsuitable for the mobilisation of slow muscle fibres (Meyer, 2006). Decreased oxygen supply and metabolite accumulation in muscle fibres can stimulate type afferents and inhibit alpha-motor neurons, thereby promoting the recruitment of fast muscle fibres to maintain muscle strength (Loenneke et al., 2010). Similar to traditional high-intensity training, low-intensity KT has similar fast muscle motor units and discharge frequency and can activate fast muscle fibres that participate in muscle activities. In the present study, the 180°/s PT improved in knee extensor (14.3% vs. 33.3%) and ankle extensor (24.2% vs. 31.1%) in CIG than the 60°/s PT. The combined intervention has more positive effects on preventing falls in older adults than a single intervention.

### 4.2 Muscular endurance

The hip and knee extension and the ankle flexion ER increased by 10.0, 10.9% and 5.7%, respectively, in CIG. The hip extension ER in WG, KG and CG at 16 weeks were compared with that in CIG. The results indicated that a single intervention did not significantly improve the ERs of the lower limb joint muscle groups in older female subjects. Appears to be some additional benefit from combined intervention above those derived from single-interventions. Compared with a single intervention, the combined intervention was more effective in improving hip extension ER in older female subjects.

By observing the effect of WBVT on joint muscle endurance in older adults, Lin et al. (2015) reported that 24 weeks of WBVT (30–40 Hz) improved the muscle endurance of the knee and ankle of older women but had no significant effect on the hip. This study found that 16 weeks of WBVT alone did not significantly improve the muscle endurance of the lower extremities in older subjects. Our results may be related to the intensity or duration of the intervention. Experiments in previous studies were performed for 24 weeks (Fachina et al., 2013; Lin et al., 2015; Castillo et al., 2018), whereas only 16 weeks were used in the present study. When on the vibration platform, WBVT caused the muscle fibres of the vibration site to have a low threshold to participate in contraction, alleviating the fatigue of the high-threshold muscle fibres (Marin et al., 2014; Castillo et al., 2018; Oliveira et al., 2021). Increasing the joint muscle strength can provide energy reserves during active and repetitive contraction movements and increase a muscle’s resistance to fatigue (Fachina et al., 2013; de Paula et al., 2021; Gonçalves et al., 2023).

The effects of KT on muscle endurance have been reported. Wilk et al. (Wilk, Krzysztofik, Filip et al., 2020a) performed a series of studies on the effects of KT on strength endurance (Wilk, Gepfert, Krzysztofik et al., 2020b; Wilk, Krzysztofik, Filip, Szkudlarek et al., 2020c). The comparison of pressurised and unpressurised designs in a single bench press revealed different levels of pressure (Wilk, Krzysztofik, Filip, Lockie et al., 2020a; Wilk, Krzysztofik, Filip et al., 2020b). Different pressurisation methods (continuous and intermittent pressurisation) (Wilk, Gepfert, Krzysztofik et al., 2020b) can increase the number of repeats, reduce the duration of muscle tone and significantly increase peak power, average power and other indicators of muscle endurance. Given that KT improves protein kinase activity in skeletal muscles and promotes the expression of the angiogenic factor gene, it improves muscle endurance (Kambič et al., 2021). By contrast, in the present study, the effect of KT was not statistically significant (Wilk, Krzysztofik, Filip, Lockie et al., 2020a; Wilk, Krzysztofik, Filip et al., 2020b), which may be related to the different intensities of compression used. During CIG hip and knee extension, the number of muscle groups decreased during 25 times of extension exercises. No approximate conclusions were derived in WG and KT, indicating that the combined intervention is more effective in improving muscle endurance. Notably, no significant change was observed in hip and knee flexor endurance possibly because of insufficient intervention time.

This study has some limitations. Firstly, the included samples were older females only and any gender difference was not analysed. Whether male subjects have similar conclusions requires further clarity. Secondly, blood indicators affecting muscle metabolism were not tested. For example, to analyze the changes in hormones and bioactive factors such as NOS and HGH associated with muscle synthesis, etc. Intrinsic mechanisms should be explored further. Finally, an intervention program with different intensities of WBVT or KT was not designed. For example, the frequency of the optimal WBVT, the intensity of the effective KT, etc. Which should be the subject of further studies.

## 5 Conclusion

Sixteen-week WBVT and KT significantly increased the knee extensor strength in older women. Compared with a single intervention, the combined intervention can better improve the muscle strength of knee extensor and ankle flexor and extensor and the muscle endurance of hip extension. Appears to be some additional benefit from combined intervention above those derived from single-interventions (Fyfe et al., 2022).

## Data Availability

The raw data supporting the conclusion of this article will be made available by the authors, without undue reservation.
