# Supplementary figures and images for: Effects of whole-body vibration training combined with KAATSU training on lower limb joint muscle strength in older women
Source: Front Physiol. 2023 Aug 29;14:1231088. doi: 10.3389/fphys.2023.1231088 (PMC10495992; doi:10.3389/fphys.2023.1231088)

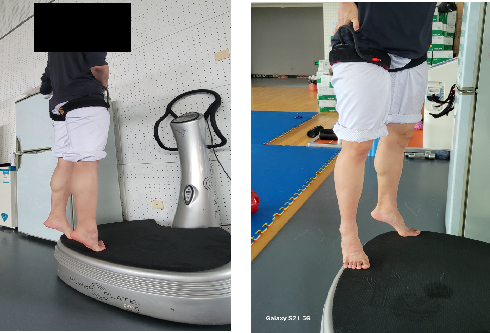

Supplement: Supplementary file 1 [file Image1.TIF]
